# Phenomapping of Patients with Primary Breast Cancer Using Machine Learning-Based Unsupervised Cluster Analysis

**DOI:** 10.3390/jpm11040272

**Published:** 2021-04-05

**Authors:** Sara Ferro, Daniele Bottigliengo, Dario Gregori, Aline S. C. Fabricio, Massimo Gion, Ileana Baldi

**Affiliations:** 1 Unit of Biostatistics, Epidemiology and Public Health, Department of Cardiac Thoracic Vascular Sciences and Public Health, University of Padova, Via Loredan 18, 35121 Padova, Italy; sara.ferro.5@gmail.com (S.F.); daniele.bottigliengo@phd.unipd.it (D.B.); dario.gregori@unipd.it (D.G.); 2 Veneto Institute of Oncology IOV-IRCCS, 35128 Padua, Italy; alinesueli.coelhofabricio@iov.veneto.it; 3 Regional Center for Biomarkers, Department of Clinical Pathology, Azienda ULSS 3 Serenissima, 30122 Venice, Italy; massimo.gion@aulss3.veneto.it

**Keywords:** primary breast cancer, prognostic factors, clustering, unsupervised learning

## Abstract

Primary breast cancer (PBC) is a heterogeneous disease at the clinical, histopathological, and molecular levels. The improved classification of PBC might be important to identify subgroups of the disease, relevant to patient management. Machine learning algorithms may allow a better understanding of the relationships within heterogeneous clinical syndromes. This work aims to show the potential of unsupervised learning techniques for improving classification in PBC. A dataset of 712 women with PBC is used as a motivating example. A set of variables containing biological prognostic parameters is considered to define groups of individuals. Four different clustering methods are used: K-means, self-organising maps, hierarchical agglomerative (HAC), and Gaussian mixture models clustering. HAC outperforms the other clustering methods. With an optimal partitioning parameter, the methods identify two clusters with different clinical profiles. Patients in the first cluster are younger and have lower values of the oestrogen receptor (ER) and progesterone receptor (PgR) than patients in the second cluster. Moreover, cathepsin D values are lower in the first cluster. The three most important variables identified by the HAC are: age, ER, and PgR. Unsupervised learning seems a suitable alternative for the analysis of PBC data, opening up new perspectives in the particularly active domain of dissecting clinical heterogeneity.

## 1. Introduction

Personalised medicine research aims to improve individual patients’ clinical outcomes through more precise treatment targeting, through the leveraging of genetic, biomarker, phenotypic, or psychosocial characteristics that distinguish a given patient from another with a similar clinical presentation [1].

The majority of studies involving patient similarity, mature enough to produce knowledge that directly informs treatment-targeting decisions, belong to the cancer domain [2]. Advances in breast cancer care are among the most paradigmatic examples of the benefits of personalised medicine research.

Breast cancer is a disease that has been thoroughly profiled on various levels, revealing high heterogeneity [3,4]. At each level, the separation of breast tumours into different groups has been used to identify disease subgroups [5,6,7], which assist in patient management.

Defining a similarity measure that can deal with the high-dimensional space of patient data is an essential step to stratify patients into clinically meaningful subgroups and enable personalised medicine.

According to a recent literature review [2], the principal methodologies used to calculate such similarities pertain to unsupervised learning.

Unsupervised learning focuses on unveiling hidden and intrinsic structures within the data and exists on a continuum with traditional statistical methods such as principal component analysis, mixture modelling, and several cluster analysis methods [8]. However, some new techniques have sparked global interest in recent years, such as deep learning [9]. Unsupervised learning is related to many branches of knowledge, such as image processing and document classification, and plays an important role in a broad range of health applications.

With the increasing possibilities to characterise the clinical, genomic, and molecular attributes of patients with a given disease, one of the promising uses of unsupervised learning is the delineation of diagnostic and prognostic subgroups to hopefully assist in the development of individualised preventive and therapeutic strategies. In this context, the term “phenomapping” [10,11] may be used. Phenomapping stems from cardiology literature and refers to the subtyping of heterogeneous clinical syndromes, with the ultimate goal of defining therapeutically homogeneous patient subclasses. Unsupervised learning finds application in oncology to discern patterns from large, complex, and noisy datasets, including genomic characterisations [12,13,14].

Despite the large variety of flexible models and algorithms for clustering available [15], K-means remains the preferred tool for most real-world applications [16], and the so-called cluster validity problem [17] receives little attention. Clustering algorithms are sensitive to the values of their parameters. Even a good clustering algorithm could produce undesirable results if parameters, particularly the number of clusters, are chosen improperly. In general, the selection of the optimal number of clusters and the evaluation of results of clustering algorithms require further attention. In this work, we illustrate and compare in terms of cluster validity different unsupervised learning methods: K-means, self-organizing maps [18] (SOM), hierarchical agglomerative clustering (HAC), and the Gaussian mixture model (GMM) [19].

The clustering algorithms are used to identify distinct subtypes of primary breast cancer (PBC) in a historical patient series, previously evaluated by traditional statistics for possible associations among covariates.

## 2. Materials and Methods

### 2.1. Motivating Example

Existing data from a study aimed at investigating the relationship between cathepsin D and steroid receptors in a cohort of women with PBC serves as the motivating example. A subgroup of 712 women was included from the original study, whose details are given elsewhere [20]. In addition to the data analysed in [20], the oestrogen-induced pS2 protein measurements were included to investigate their relationship with PBC further. The following information on biological and pathological prognostic parameters was extracted from the database for each patient: age, menopausal status, oestrogen receptor (ER), progesterone receptor (PgR), pS2 protein, cathepsin D, histological type, number of positive lymph nodes, and tumour size diameters.

### 2.2. Clustering Algorithms

The present section describes the clustering algorithms that were considered in the study. Table 1 summarises the main characteristics of the algorithms, along with their advantages and disadvantages.

#### 2.2.1. K-Means Clustering

K-means clustering is one of the simplest and most popular unsupervised machine learning algorithms [21]. The algorithm’s goal is to subset the observations of a dataset into separate K clusters, whose number is chosen a priori. The method assigns each data point to one of K clusters based on the features in the dataset, using as a criterion a dissimilarity measure based on the Euclidean distance. The assignment of each observation to a cluster is performed through the following steps: (1) for each cluster, a centroid is defined that is the mean vector of the variables in the cluster; (2) for each observation, the Euclidean distance between the observation and the centroid is computed. The observation is then assigned to the cluster with the smaller distance from the centroid to minimise the within-cluster variance; (3) steps 1–2 are iteratively repeated until the clusters’ assignment of observations does not change. The K-means clustering algorithm has been successfully applied in breast cancer research [25,26,27].

#### 2.2.2. Self-Organising Maps (SOM) Clustering

A SOM or self-organising feature map (SOFM) is a type of artificial neural network (ANN) that is trained using unsupervised learning to produce a low-dimensional, discretised representation of the input space of the training samples, called a map. It is, therefore, a method used to make dimensionality reductions. SOMs differ from other ANNs, as they apply competitive learning instead of error-correction learning, i.e., they use a neighbourhood function to preserve the topological properties of the input space.

SOMs can be viewed as a constrained version of K-means, in which the original high-dimensional objects are constrained to map onto a one- or two-dimensional coordinate system. This allows the visualisation of similar clusters close to each other and thus helps the user (although a posteriori) choose the best number of clusters. Like K-means, it is designed for quantitative variables and the choice of (weighted) Euclidean distance as a dissimilarity measure. SOM methods have been used in a few medical studies [28,29,30].

#### 2.2.3. Hierarchical Agglomerative Clustering (HAC)

HAC algorithms partition the data into clusters with a hierarchical structure, as the name suggests. Unlike K-means and SOM approaches, they do not require the user to pre-specify the number of clusters into which data must be grouped. These methods only require a specification of a similarity measure that is used to allocate observations into subgroups. The output of these methods is often represented as a hierarchical tree, where the leaves represent units grouped into clusters, and the roots represent the superclusters containing all the objects. Hierarchical agglomerative clustering is a bottom-up technique that, starting at the bottom of the clustering structure, recursively merges pairs of clusters into a single cluster at each structural level. The algorithm’s implementation can be summarised as follows: (1) a dissimilarity measure is computed for two groups of observations; (2) the most similar groups are merged into a single cluster; (3) steps 1–2 are repeated until all the observations belong to a single cluster. Hierarchical clustering has been widely applied in the medical literature, aiding the identification of subgroups of breast cancer patients with meaningful profiles [31,32].

#### 2.2.4. Gaussian Mixture Model (GMM)

GMMs are one of the most popular model-based clustering approaches. They assume that observations of a dataset are generated from a mixture of K Gaussian distributions, whose density is governed by a vector of means (one for each distribution), a covariance matrix, and a vector of mixing proportions. The parameters of the distributions are generally unknown, and they have to be estimated. A maximum likelihood estimator (MLE) is usually employed to estimate the parameters of the distribution, using the expectation-maximisation (EM) algorithm [30,31]. Most applications assume that all components have the same parameters of the Gaussians, i.e., the same covariance matrix and the same mean for all the K distributions. This is not the general case, and the mixture of Gaussian distributions can be parametrised more flexibly by choosing different geometric structures through the eigenvalue decomposition of the covariance matrix. More technical details on the implementation of GMMs can be found elsewhere [19]. As for K-means and SOMs, the number of K distributions (i.e., the number of clusters) must be pre-specified by the user, and they can be applied for continuous variables only. The main advantage of GMMs is that they can estimate the probability that each observation belongs to the K clusters. The observations can be then assigned to the clusters with the highest membership probability. A limited number of studies used GMMs to define breast cancer patients’ groups based on their characteristics [33].

### 2.3. Statistical Analysis

Continuous variables are represented with I quartile, median, and III quartile. Categorical variables are represented with percentages (absolute numbers). The following characteristics of the patients were considered in the clustering models: age, level of oestrogen receptor in mg of cytosol protein (fmol/mg CP), level of PgR (fmol/mg CP), pS2 protein level (pmol/mg CP), and cathepsin D levels (pmol/mg CP). Only the patients’ quantitative characteristics were used since the algorithms considered in the present study deal with continuous variables. Tumour size was excluded from the analysis given the high percentage of missing data (49%). Unsupervised techniques were internally validated, i.e., the models’ goodness-of-fit was evaluated using only the case study’s data, without testing it on external data. Internal validation assesses the performance of a clustering algorithm by comparing the structure of the clusters identified in the data and their relation to each other. The internal validation procedure assumes that the algorithm has found “good” clusters according to two concepts: compactness and separation. The former evaluates how close the observations are in the cluster, whereas the latter assesses how dissimilar the clusters identified by the algorithm are. Internal validation is considered much more efficient and realistic than external validation, as in many realistic scenarios it is hard to obtain assumed references from outside [34]. In the present study, internal validation was carried out using the clValid function of the homonym R package [35].

The explored range of the number of clusters was from 2 to 18 via the parameter nClust. The optimal number of clusters was chosen according to the following measures: connectivity, Dunn index and silhouette width. Briefly, the connectivity index measures the trade-off between how close the units in the same cluster are and how well-separated the units belonging to different clusters are. Lower connectivity values are associated with better clustering performances. The Dunn index is the ratio between the minimum pairwise inter-cluster distance and the maximum intra-cluster distance (also called the cluster diameter). Higher Dunn index values denote better algorithm performance, since the clusters are well-identified when the minimum pairwise inter-cluster distance and the maximum intra-cluster difference is low. The silhouette width measures the average distance between clusters, and higher values are associated with the better performance of the algorithm. Moreover, the clustering technique with the best overall internal validation was determined with a rank aggregation procedure based on a cross-entropy Monte Carlo algorithm and the default measure of distance (Spearman) to compute the distances between any two ordered lists as implemented in the optCluster R package [36]. For each algorithm, the strength of the variables in the definition of the clusters was assessed. Regarding K-means, a procedure based on the misclassification rate for assignment to the clusters due to a permutation of the variables’ values was used through the FeatureImpCluster function of the homonym R package [37]. For SOM, a Bayesian approach that evaluates each variable’s importance based on variance was considered [38]. It was evaluated with the package popsom and the map.significance function with default parameters [39]. For HAC, the R package named FactoMineR and the function HCPC, which performs the hierarchical clustering on the principal components, was used [40]. It gives as output the sequence of the most important variables for the clustering. A greedy variable importance algorithm with a forward direction was used for the GMM [41].

The clusters’ agreement identified with the four methods was evaluated using Meila’s variation of information (VI) distance, an index that measures the similarity between clusters obtained with two different methods [42]. Lower VI values denote higher similarity between clusters obtained with two different algorithms.

The analysis was performed using the R software for statistical computing (version 4.0.2) [43]. The internal validation was performed using the clValid package [35]. The SOM algorithm was implemented using the kohonen package [44], and the mclust R package [45] was used for the GMM. Variable importance was assessed with FeatureImpClusters, popsom, FactoMineR, and clustvarsel packages for K-means, SOM, HAC, and the GMM, respectively. Meila’s VI was computed using the package fpc [46].

## 3. Results

Overall, 712 women with PBC were considered in the analysis. Table 2 shows the distributions of the characteristics of the patients in the sample. The median age of the patients is 62 years. Median values of ER and PgR are 49.5 fmol/(mg CP) and 42.0 fmol/(mg CP), respectively. The distributions of pS2 protein and cathepsin D have median values of 6.6 pmol/(mg CP) and 39.2 pmol/(mg CP), respectively. The majority of patients (73.18%) are postmenopausal. The prevalent histological type is the infiltrating ductal (72.21%). More than half of patients were node-negative (54.5%). Median tumour diameter is 2.00 cm.

The internal validity indexes values are reported in Table 3. The values refer to the performances of the algorithm with the optimal number of clusters (in brackets). Overall, two clusters were identified as the optimal number by all the methods except for K-means and SOM according to the Dunn index (18 and 6 clusters, respectively) and the silhouette measure (4 clusters for both algorithms). The clusters obtained with the HAC algorithm showed the highest performances in terms of internal validity according to all three measures, whereas the poorest performances were observed for the clusters obtained with the GMM. The rank aggregation procedure results are in line with those observed for connectivity, Dunn, and silhouette indexes, i.e., the best performing algorithm was HAC with two clusters.

Since the best performances were observed with two clusters for all the algorithms according to the majority of the indexes, the values of Dunn and silhouette measures were also assessed for K-means and SOM with two clusters. Regarding K-means, a Dunn index equal to 0.011 and a silhouette index equal to 0.298 were observed, and they were similar to those obtained with the optimal number of clusters. Regarding SOM, the results were in line with those observed with the optimal number of clusters, i.e., 0.011 and 0.299 for Dunn and silhouette, respectively.

**Figure 1:**
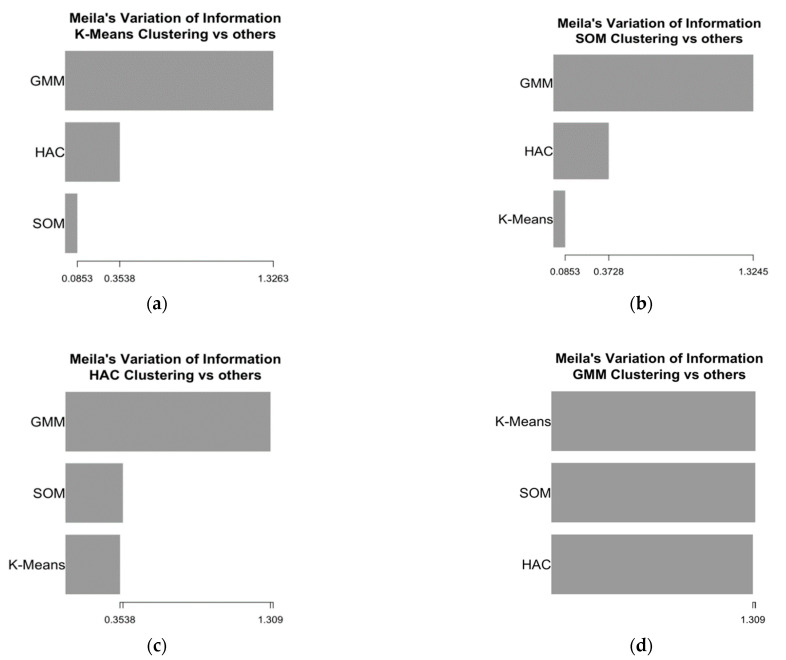
Meila’s variation of information (VI) of similarity for the two clusters obtained with four algorithms. (**a**) Meila’s VI for K-means; (**b**) Meila’s VI for SOM; (**c**) Meila’s VI for HAC; (**d**) Meila’s VI for the GMM.

The performances obtained with two clusters were good or optimal for all the algorithms. For this reason, we addressed the aspect of assessing the similarity of the clusters obtained with different methods, a task that would not be possible to achieve had the algorithms returned a different number of clusters. The similarity of the clusters was evaluated in terms of pairwise agreement with Meila’s VI, i.e., how two algorithms agreed in terms of grouping observations in the same cluster. Meila’s VI values for all pairs of algorithms are depicted in Figure 1.

The highest similarity was observed for the clusters obtained with K-means and SOM, which had the lowest Meila’s VI. A similar agreement was also observed between the pairs K-means–HAC and SOM–HAC, whereas the clusters obtained with the GMM showed the lowest similarity with those obtained with the other methods. The distributions of the variables used to define the clusters for the groups of patients identified by the algorithms are shown in Table 4.

**Figure 2:**
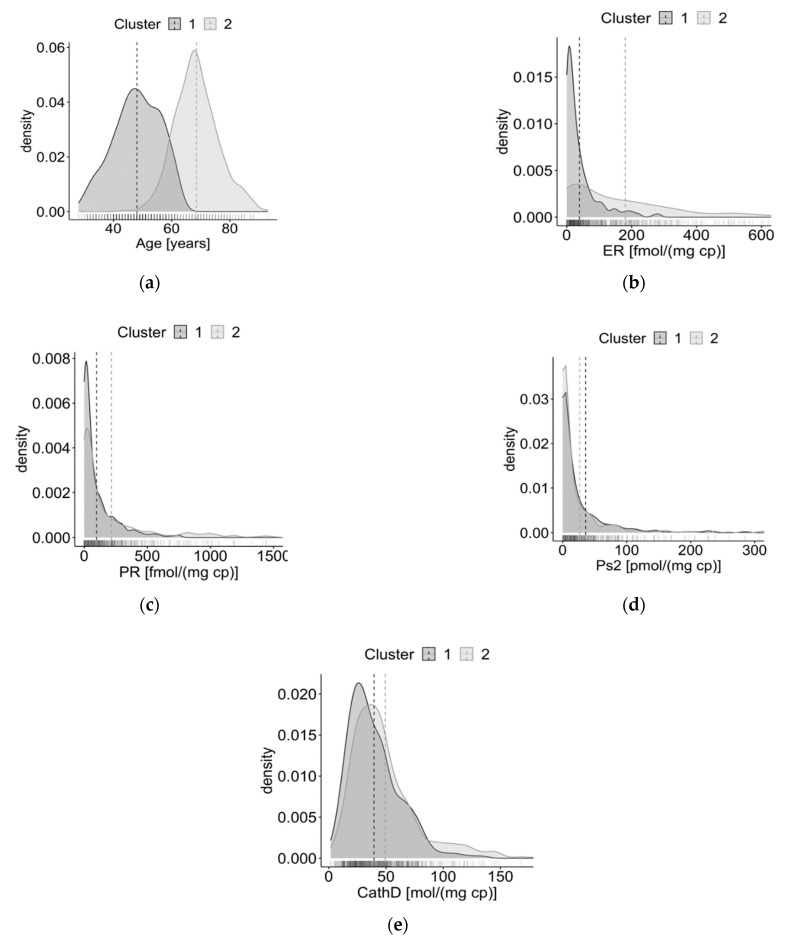
Density plots of the distributions of the variables in the two clusters obtained with the HAC algorithm. (**a**) Distributions of age; (**b**) distributions of oestrogen receptor (ER); (**c**) distributions of progesterone receptor (PgR); (**d**) distributions of pS2 protein; (**e**) distributions of cathepsin D.

Overall, patients in the second clusters are older, have higher values of ER, PgR, and cathepsin D, and lower levels of pS2 protein (except when using the GMM algorithm). In line with Meila’s VI, the distributions observed for the GMM are less similar than those observed for the other algorithms. The density plots of the variables in the clusters obtained with HAC, i.e., the algorithm that achieved the best performances, are also depicted in Figure 2. The variables’ distributions by clusters identified by the other algorithms are shown in the Supplementary material.

To provide a more comprehensive description of the groups of patients for the best partition of the data, the variables that were not included in the clustering algorithms are represented in Table 5, along with the distributions of the variables used to define the clusters.

The variable importance procedure for K-means found that age was the most important variable, followed by ER, cathepsin D, PgR, and pS2 protein. Regarding SOM, age was the most important, followed by cathepsin D, ER, PgR, and pS2 protein. HAC method for variable importance ranked the subjects’ characteristics in the following order: age, ER, PgR, cathepsin D, and pS2 protein. For the GMM, the importance ranking had the following order: ER, PgR, pS2 protein, cathepsin D, and age.

## 4. Discussion

In the present study, four unsupervised learning techniques were compared to identify subgroups of women with PBC in a historical patient series. K-means and HAC are among the most frequently used clustering methods to investigate patient similarity, whereas SOM and the GMM are more sophisticated and rarely applied to this purpose [2].

This study illustrates how these methods should be implemented and could open up new perspectives in the particularly active domain of dissecting PBC heterogeneity.

The importance of three methodological issues emerges. The first is measuring performance through internal validity indexes based on the clustering’s intrinsic statistical properties. The second is comparing partitions by measuring the amount of information lost and gained in changing from one clustering method to another. The last is to rank variables by the degree to which they help to cluster patients into each cluster (i.e., the variable importance).

Two main subgroups of women with PBC were identified by the algorithm that had the best internal validation, i.e., HAC. The number of clusters (*k* = 2) that led to the best performance was consistent according to most indexes for all the other methods. Moreover, all the algorithms showed a high degree of similarity except for the GMM, which was the method that had the poorest performance.

According to the best performing algorithm, the identified groups were strongly differentiated in terms of the characteristics used to perform clustering: one group of women with PBC was older, had higher levels of ER and PgR, higher values of cathepsin D, and lower values of pS2 protein. These data are in accordance with evidence showing that breast tumours in elderly women display high rate of ER and PgR positivity [47,48], and that, in general, the elderly population has demographic and biological characteristics distinct from their younger counterparts, which may explain the indolent nature of breast cancer in postmenopausal women [48]. As concerns cathepsin D, its high levels are significantly associated with the ER-positive and PgR-positive status of breast cancer [20,49], which might be expected as cathepsin D expression is under oestrogen control in ER-positive breast carcinomas [50]. Furthermore, the observation of lower levels of pS2 in the older group could be justified by the fact that pS2-positive protein status has only been associated with ER-positive status in patients aged 50 years or less [51]. Indeed, previous studies showed that tumour pS2 concentration tends to be lower in postmenopausal patients [52,53], whereas cathepsin D tumour level does not show significant variation between pre- and postmenopausal patients [53]. The agreement between the literature evidence and the present study’s findings reinforces the capability of HAC in identifying strongly differentiated groups.

As acknowledged by [4], these findings suggest that a novel classification for PBC should rely on subgroups with a meaningful phenotypic differentiation and should be robust to different unsupervised techniques. The recognition of a meaningful phenomapping should be made collegially, i.e., based on the results of a set of methods.

Although the GMM has been successfully applied in the molecular stratification of patients with breast cancer [33], it failed to recover the cluster structure identified by HAC, SOM, and K-means. A possible explanation is that model-based clustering assumes that the underlying model is correctly specified, and each data cluster can be viewed as a sample from a mixture component. In real-world data, the true distribution is rarely known and further, data may be contaminated by outliers from a distribution different from the Gaussian, resulting in a mis-specified model and biased results [24].

The results of the variable importance assessment highlight that for the best performing model (i.e., HAC) age, ER, and PgR levels are the most critical factors in the groups’ definition. Interestingly, the HAC algorithm identified cathepsin D as an important marker for classifying women with PBC. Moreover, the variables’ importance assessment of all the algorithms ranked cathepsin D as one of the most important clustering variables.

Based on the subgroups identified by the best method, the variation of the variables (i.e., menopausal status, histological type, number of positive lymph nodes, and cancer diameter) that were not used by the algorithms was investigated. The potential separation of their distributions in the identified clusters might suggest that the subset of characteristics considered for the analysis is good proxy for a wide range of other variables. This may improve the interpretation of the phenotypic mechanism underlying PBC, leading to a more intuitive representation of the disease, which has a very complex characterisation, with the use of a defined subset of variables.

Our study suffers from various limitations, namely the relatively small number of patients’ characteristics and the retrospective nature of the study. Besides, our results could not be validated on an external cohort. The clustering performances were assessed only by internal validation. Indeed, we could not compare the subgroups obtained from our classification with the true ones to calculate the accuracy of the classification, since the “ground-truth” PBC subgroups were unknown in the evaluated historical patient series. Furthermore, given our low-dimensional setting, we did not consider common visualization methods used in clustering analysis, such as t-SNE plots based on PCA [54], to aid the interpretation of the results. Future research is needed to study how graphical tools can enhance the interpretation of the algorithms.

From a biological point of view, the two major groups of breast cancer at the histopathological level are the ER-positive and ER-negative tumours, encompassing all other molecular subgroups. Further, at the gene expression level, five main subgroups have been identified and combining gene expression with copy number data further refined breast cancer into integrated subgroups with different genomic and transcriptomic profiles and prognosis [3]. However, heterogeneity persists in biological features within PBC subtypes, highlighting the need to improve the taxonomy [4].

Further research studies are needed to better investigate the dependencies in a multidimensional and multidisciplinary problem, aiding in the understanding of underlying characteristics and dynamics that act in PBC disease.

The use of unsupervised learning applied to PBC data is a promising approach to improving PBC classification and the definition of therapeutic patient subclasses.

## Figures and Tables

**Table 1:** Summary of the main characteristics of the algorithms, along with their pros and cons.

Algorithm	Summary Features	Pros	Cons
K-means [21]	Probably the most popular clustering algorithm Dissimilarity measure based on Euclidean distance The number of clusters must be pre-specified	Simple implementation Implemented in many software Low-computational costs	Might not be robust to outliers and skewed distributions Difficulties to identify complex relationships in the data Handles only continuous variables Deterministic clustering
Self-organizing maps (SOM) [22]	Based on artificial neural network (ANN) A constrained version of K-means The number of clusters must be pre-specified	Suitable for high-dimensional data Simple to implement and for adaptation to many situations	Deterministic clustering Handles only continuous variables Can be unstable when clusters are difficult to identify
Hierarchical agglomerative clustering (HAC) [23]	Cluster with a hierarchical structure Graphically represented as a hierarchical tree No pre-specification of the number of clusters	Simple implementation Easy to interpret Suitable for identifying subgroups of patients	High computational costs with complex clustering structures Difficult to identify the right number of clusters with large datasets Handles only continuous variables Deterministic clustering
Gaussian mixture model (GMM) [24]	Model-based clustering approach Data generated from a mixture of normal distributions The number of clusters must be pre-specified	Simple implementation Implemented in many software Estimates the probability of being in each cluster	Might not be robust to outliers and skewed distributions Handles only continuous variables High computational costs with large datasets and complex clustering structures

**Table 2:** Distributions of the variables in the sample. Continuous variables are represented with median values (1st quartile–3rd quartile). Categorical variables with percentages. N is the number of non-missing observations for each variable.

Characteristic	N	Statistics
Age (years)	712	62 (51–69)
ER (fmol/(mg CP))	712	49.5 (9.0–172.8)
PgR (fmol/(mg CP))	712	42.0 (12.0–182.0)
PS2 protein (pmol/(mg CP))	712	6.6 (0.9–26.5)
Cathepsin D (pmol/(mg CP))	712	39.2 (25.6–55.6)
Menopausal status:		
Pre-menopausal	158	22.2%
Peri-menopausal (no menses <2 years)	33	4.6%
Post-menopausal (no menses >2 years)	521	73.2%
Histological type:		
Intraductal	38	5.3%
Infiltrating ductal	514	72.2%
Infiltrating ductal and infiltrating lobular	31	4.4%
Infiltrating ductal with different aspects	16	2.2%
Non-infiltrating lobular	10	1.4%
Infiltrating lobular	54	7.6%
Medullary	9	1.3%
Multi-centred infiltrating ductal	7	1.0%
Multi-centred infiltrating lobular	1	0.1%
Others	32	4.5%
N. positive lymph nodes:		
0	382	53.6%
1	112	15.7%
2	55	7.7%
>2	163	23%
Cancer diameter (cm)	349	2.00 (1.50–3.00)

**Table 3:** Values of indexes that were used to assess the internal validity of the clustering algorithms. The values refer to the score obtained with the optimal number of clusters, which is reported in brackets.

Algorithm	Connectivity	Dunn	Silhouette
K-means	76.992 (2)	0.020 (18)	0.335 (4)
SOM	80.090 (2)	0.012 (6)	0.338 (4)
HAC	2.930 (2)	0.719 (2)	0.740 (2)
GMM	390.610 (2)	0.011 (2)	0.123 (2)

**Table 4:** Distributions of the variables in the two clusters obtained with the four algorithms (size of cluster 1, size of cluster 2). Variables are represented with median values (1st quartile–3rd quartile).

		Method			
Variable	Cluster	K-Means (284, 428)	SOM (280, 432)	HAC (277, 435)	GMM (279, 433)
Age (years)	Cluster 1	48 (43–54)	48 (43–54)	48 (43–55)	62 (51–69)
	Cluster 2	68 (64–73)	68 (63–73)	68 (64–73)	63 (51–64)
ER (fmol/(mg CP))	Cluster 1	20.0 (5.0–50.0)	20.0 (5.0–48.0)	20.0 (5.0–50.0)	22.0 (4.0–80.0)
	Cluster 2	115.0 (23.8–254.3)	115.0 (24.0–256.0)	112.0 (22.5–254.5)	152.0 (40.5–336.0)
PgR (fmol/(mg CP))	Cluster 1	36.0 (9.0–144.0)	36.0 (9.0–147.0)	35.0 (9.0–128.0)	19.0 (7.0–50.0)
	Cluster 2	44.5 (12.8–215.5)	44.0 (12.5–215.0)	48.0 (13.0–232.0)	240.0 (97.0–464.0)
pS2 protein (pmol/(mg CP))	Cluster 1	8.3 (1.0–31.2)	7.8 (1.0–31.0)	6.7 (0.9–29.9)	2.9 (0.5–11.8)
	Cluster 2	5.9 (0.9–22.9)	6.0 (0.9–23.0)	6.5 (0.9–24.2)	24.9 (5.2–72.7)
Cathepsin D (pmol/(mg CP))	Cluster 1	35.1 (23.6–50.0)	35.3 (23.5–50.2)	34.5 (23.1–50.5)	34.7 (23.6–49.0)
	Cluster 2	41.4 (27.4–60.0)	41.2 (27.2–59.9)	42.0 (28.5–59.3)	45.7 (31.7–67.0)

**Table 5:** Distributions of the variables in the two clusters obtained with HAC. Continuous variables are represented with median values (1st quartile–3rd quartile). Categorical variables with percentages (absolute numbers).

Characteristic	Cluster 1 (*N* = 277)	Cluster 2 (*N* = 435)
Age (years)	48 (43–55)	68 (63.5–73)
Menopausal status:		
Pre-menopausal	54.9% (152)	1.4% (6)
Peri-menopausal (no menses <2 years)	11.5% (32)	0.2% (1)
Post-menopausal (no menses >2 years)	33.6% (93)	98.4% (428)
ER (fmol/(mg CP))	20 (5–50)	112 (22.5–254.5)
PgR (fmol/(mg CP))	35 (9–128)	48 (13–232)
pS2 protein (pmol/(mg CP))	6.7 (0.9–29.9)	6.5 (0.9–24.2)
Cathepsin D (pmol/(mg CP))	34.5 (23.1–50.5)	42 (28.5–59.3)
Histological type:		
Intraductal	8.7% (24)	3.2% (14)
Infiltrating ductal	69.0% (191)	74.3% (323)
Infiltrating ductal and infiltrating lobular	5.1% (14)	3.9% (17)
Infiltrating ductal with different aspects	1.8% (5)	2.5% (11)
Non-infiltrating globular	0.0% (0)	2.3% (10)
Infiltrating globular	7.2% (20)	7.8% (34)
Medullary	1.4% (4)	1.1% (5)
Multi-centred infiltrating ductal	1.4% (4)	0.7% (3)
Multi-centred infiltrating globular	0.4% (1)	0.0% (0)
Others	5.0% (14)	4.2% (18)
N. positive lymph nodes		
0	55.6% (154)	52.4% (228)
1	16.6% (46)	15.2% (66)
2	8.3% (23)	7.3% (32)
>2	19.5% (54)	25.1% (59)
Cancer diameter (cm)	2 (1.5–2.5)	2 (1.5–3)

## Data Availability

The data presented in this study are available on request from the corresponding author. The data are not publicly available due to privacy restrictions.

## Supplementary Materials

The following are available online at https://www.mdpi.com/article/10.3390/jpm11040272/s1. Figure S1: Density plots of the distributions of the variables in the two clusters obtained with the K-means algorithm: (a) Distributions of age; (b) Distributions of ER; (c) Distributions of progesterone; (d) Distributions of ps2; (b) Distributions of cathepsin D. Figure S2: Density plots of the distributions of the variables in the two clusters obtained with the SOM algorithm: (a) Distributions of age; (b) Distributions of ER; (c) Distributions of PgR; (d) Distributions of ps2; (b) Distributions of cathepsin D. Figure S3: Density plots of the distributions of the variables in the two clusters obtained with the GMM algorithm: (a) Distributions of age; (b) Distributions of oestrogen; (c) Distributions of PgR; (d) Distributions of ps2; (b) Distributions of cathepsin D. R code with simulated data.
