# Supplementary material for: Phenomapping of Patients with Primary Breast Cancer Using Machine Learning-Based Unsupervised Cluster Analysis
Source: J Pers Med. 2021 Apr 5;11(4):272. doi: 10.3390/jpm11040272 (PMC8067194; doi:10.3390/jpm11040272)
Supplement: Supplementary file 1 [file jpm-11-00272-s001.pdf]

Journal of *Personalized Medicine*

MDPI

1. Density Plots of the Distributions

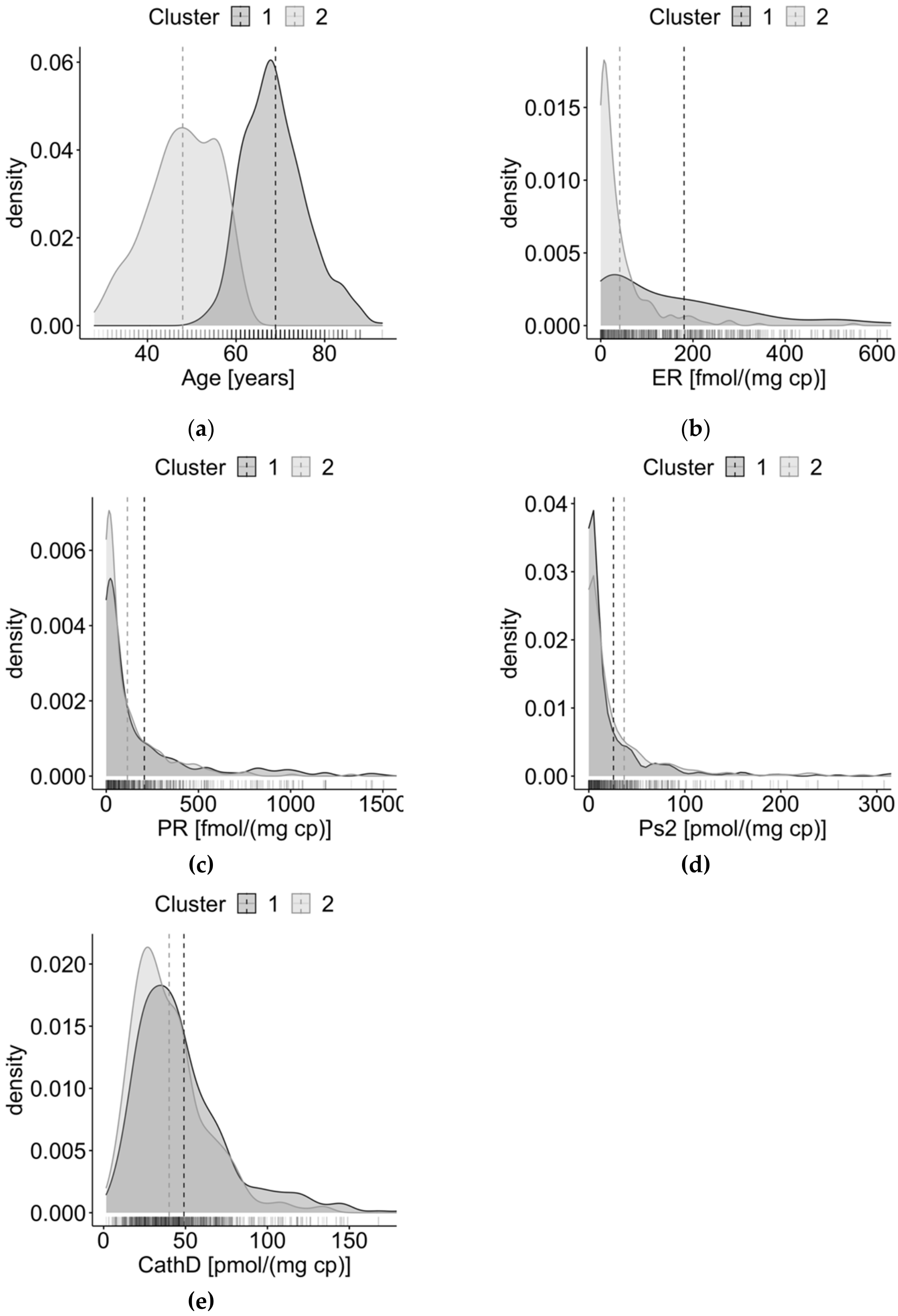

Figure S1. Density plots of the distributions of the variables in the two clusters obtained with the K-means algorithm. (a) Distributions of age; (b) Distributions of ER; (c) Distributions of PgR; (d) Distributions of ps2; (b) Distributions of cathepsin D.

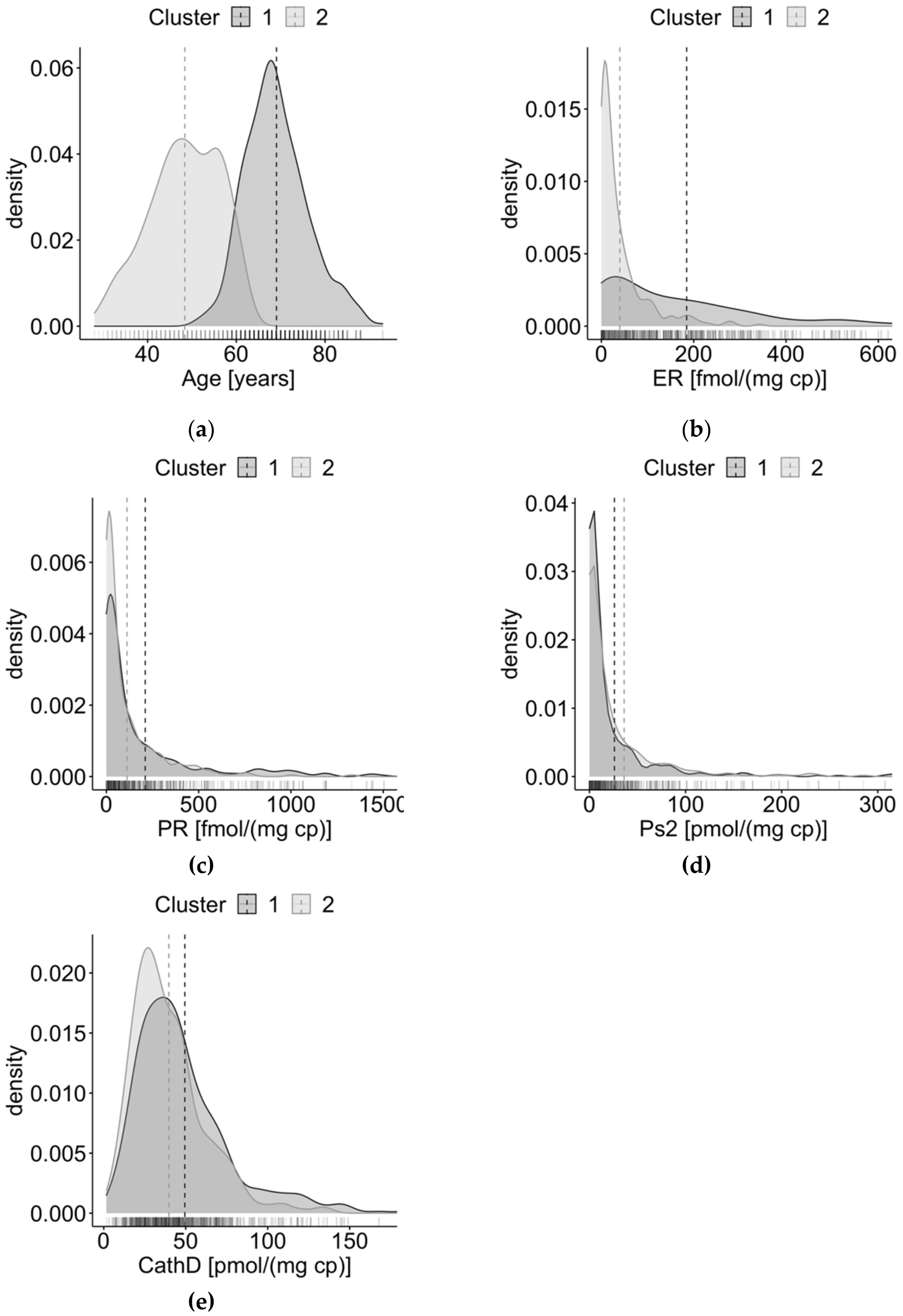

Figure S2. Density plots of the distributions of the variables in the two clusters obtained with the SOM algorithm. (a) Distributions of age; (b) Distributions of ER; (c) Distributions of PgR; (d) Distributions of ps2; (b) Distributions of cathepsin D.

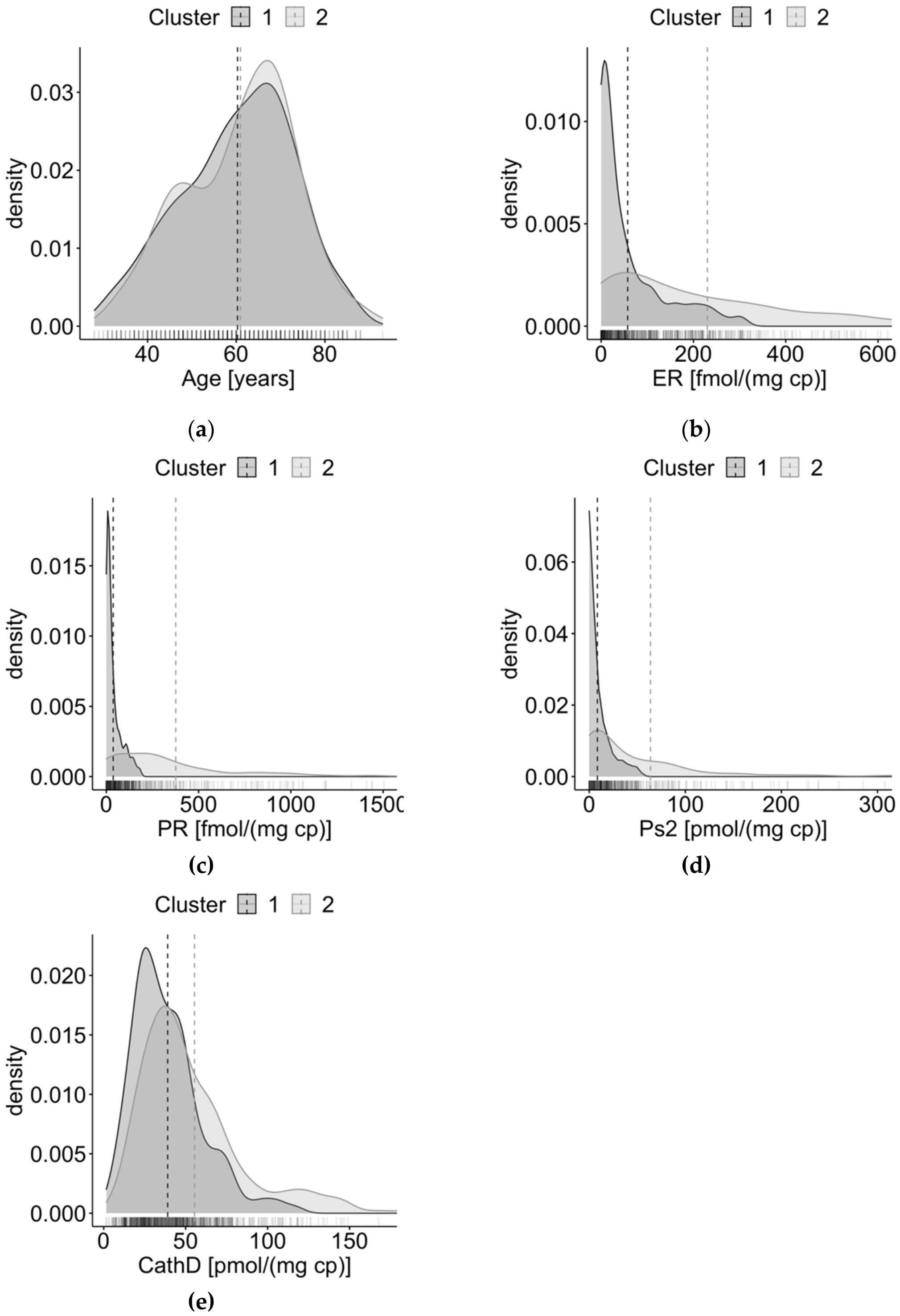

Figure S3. Density plots of the distributions of the variables in the two clusters obtained with the GMM algorithm. (a) Distributions of age; (b) Distributions of ER; (c) Distributions of PgR; (d) Distributions of ps2; (b) Distributions of cathepsin D.

2. Code

```
install.packages(c("RColorBrewer","readxl","latex2exp","corrplot","clValid","optClus-
ter","FeatureImpCluster","popsom","FactoMineR","fpc"))
```

```
library(RColorBrewer)
library(ggpubr)
library(readxl)
library(latex2exp)
library(corrplot)
library(clValid)
library(optCluster)
library(FeatureImpCluster)
library(popsom)
library(FactoMineR)
if(!require(devtools)) install.packages("devtools")
devtools::install_github("cran/clustvarsel")
library(clustvarsel)
library(fpc)


preproc <-function(x){
  x <- as.vector(x)
    i = 1
    for(i in c(1:length(x))){
       if(x[i]<0){
         x[i]=0.00
       }
      i = i+1
    }
  x}

# Simulated data

n_samples = 712
Age <- rnorm(n_samples, 48.04, 7.99)+rnorm(n_samples, 68.51, 7.48)
ER <- rnorm(n_samples,38.86,52.49)+rnorm(n_samples,180.10,218.74)
PR <- rnorm(n_samples,97.80, 139.18) + rnorm(n_samples,215.98, 404.65)
Ps2 <- rnorm(n_samples, 35.81, 158.89) + rnorm(n_samples,26.53, 58.69)
CathD <- rnorm(n_samples,39.47,22.06) + rnorm(n_samples,49.24, 33.34)

numericData <- cbind(Age,ER,PR,Ps2,CathD)
numericData <- apply(numericData, 2, preproc)
summary(numericData)
numericDataScaled <- scale(numericData, center = F, scale = apply(numericData,2,max))

source("clValid-Classes.R")
source("clValid-functions.R")
source("clValid-internal.R")
source("clValid-Methods.R")

minNClust <- 2
maxNClust <- 18

clmethods <- c("model","hierarchical","kmeans","som")
internValid <- clValid(numericDataScaled, nClust = minNClust:maxNClust,
                  clMethods = clmethods, validation = "internal", verbose = TRUE,
method = "ward", maxitems = nrow(numericDataScaled))
optimalScores(internValid)
```

```
internValidKMeans <- measures(internValid)[,,"kmeans"]
internValidKMeans <- as.data.frame(t(internValidKMeans))
internValidHAC <- measures(internValid)[,,"hierarchical"]
internValidHAC <- as.data.frame(t(internValidHAC))
internValidGMM <- measures(internValid)[,,"model"]
internValidGMM <- as.data.frame(t(internValidGMM))
internValidSOM <- measures(internValid)[,,"som"]
internValidSOM <- as.data.frame(t(internValidSOM))

which.min(measures(internValid)[,,"kmeans"]["Connectivity",])
which.min(measures(internValid)[,,"som"]["Connectivity",])
which.min(measures(internValid)[,,"hierarchical"]["Connectivity",])
which.min(measures(internValid)[,,"model"]["Connectivity",])

min(measures(internValid)[,,"kmeans"]["Connectivity",])
min(measures(internValid)[,,"som"]["Connectivity",])
min(measures(internValid)[,,"hierarchical"]["Connectivity",])
min(measures(internValid)[,,"model"]["Connectivity",])

which.max(measures(internValid)[,,"kmeans"]["Dunn",])
which.max(measures(internValid)[,,"som"]["Dunn",])
which.max(measures(internValid)[,,"hierarchical"]["Dunn",])
which.max(measures(internValid)[,,"model"]["Dunn",])

max(measures(internValid)[,,"kmeans"]["Dunn",])
max(measures(internValid)[,,"som"]["Dunn",])
max(measures(internValid)[,,"hierarchical"]["Dunn",])
max(measures(internValid)[,,"model"]["Dunn",])

which.max(measures(internValid)[,,"kmeans"]["Silhouette",])
which.max(measures(internValid)[,,"som"]["Silhouette",])
which.max(measures(internValid)[,,"hierarchical"]["Silhouette",])
which.max(measures(internValid)[,,"model"]["Silhouette",])

max(measures(internValid)[,,"kmeans"]["Silhouette",])
max(measures(internValid)[,,"som"]["Silhouette",])
max(measures(internValid)[,,"hierarchical"]["Silhouette",])
max(measures(internValid)[,,"model"]["Silhouette",])

numericDataScaledDf <- as.data.frame(numericDataScaled)
optVal <- optCluster(numericDataScaledDf, nClust = minNClust:maxNClust,
                     clMethods = clmethods, validation = "internal",
           hierMethod = "ward", metric = "euclidean", clVerbose = TRUE)
topMeth <- topMethod(optVal)

kOpt=2

dist<- dist(numericDataScaled,method="euclidian")
dist <- dist^2
hClustOpt = hclust(dist,method="ward.D")

classifOptHac <- cutree(hClustOpt,k=2)
```

```
classifOptHacClN <- as.factor(classifOptHac)
summary(classifOptHacClN)

indexHacKOpt <- classifOptHac

class(numericData)
numericDataDf <- as.data.frame(numericData)

datasetIdxHacKOpt <- cbind(numericDataDf,indexHacKOpt)
colnames(datasetIdxHacKOpt)<-c(names(numericDataDf),"Cluster")
datasetIdxHacKOpt$Cluster <- as.factor(datasetIdxHacKOpt$Cluster)

p<-ggdensity(datasetIdxHacKOpt, x = "Age",
             add = "mean", rug = TRUE,
             color = "Cluster", fill = "Cluster",
             palette = c("grey10","grey60"), alpha=0.25, xlab = "Age [years]")
ggpar(p,xlim = c(min(datasetIdxHacKOpt$Age),max(datasetIdxHacKOpt$Age)),
        font.submain = c(20),
        font.x = c(20),
        font.y = c(20),
        font.title = c(20),
        font.subtitle = c(20),
        font.tickslab = c(20),
        font.legend = c(20))

p<-ggdensity(datasetIdxHacKOpt, x = "ER",
             add = "mean", rug = TRUE,
             color = "Cluster", fill = "Cluster",
             palette = c("grey10","grey60"), alpha=0.25, xlab = "ER [fmol/(mg cp)]")
ggpar(p,xlim = c(min(datasetIdxHacKOpt$ER),600),
        font.submain = c(20),
        font.x = c(20),
        font.y = c(20),
        font.title = c(20),
        font.subtitle = c(20),
        font.tickslab = c(20),
        font.legend = c(20))

p<-ggdensity(datasetIdxHacKOpt, x = "PR",
             add = "mean", rug = TRUE,
             color = "Cluster", fill = "Cluster",
             palette = c("grey10","grey60"), alpha=0.25, xlab = "PR [fmol/(mg cp)]")
ggpar(p,xlim = c(min(datasetIdxHacKOpt$PR),1500),
        font.submain = c(20),
        font.x = c(20),
        font.y = c(20),
        font.title = c(20),
        font.subtitle = c(20),
        font.tickslab = c(20),
        font.legend = c(20))

p<-ggdensity(datasetIdxHacKOpt, x = "Ps2",
             add = "mean", rug = TRUE,
             color = "Cluster", fill = "Cluster",
```

```
            palette = c("grey10","grey60"), alpha=0.25, xlab = "Ps2 [pmol/(mg cp)]")
ggpar(p,xlim = c(min(datasetIdxHacKOpt$Ps2),300),
        font.submain = c(20),
        font.x = c(20),
        font.y = c(20),
        font.title = c(20),
        font.subtitle = c(20),
        font.tickslab = c(20),
        font.legend = c(20))

p<-ggdensity(datasetIdxHacKOpt, x = "CathD",
            add = "mean", rug = TRUE,
            color = "Cluster", fill = "Cluster",
            palette = c("grey10","grey60"), alpha=0.25, xlab = "CathD [mol/(mg cp)]")
ggpar(p,xlim = c(min(datasetIdxHacKOpt$CathD),0.60*max(datasetIdxHacKOpt$Cath
D)),
        font.submain = c(20),
        font.x = c(20),
        font.y = c(20),
        font.title = c(20),
        font.subtitle = c(20),
        font.tickslab = c(20),
        font.legend = c(20))

kMeans_kOpt <- kmeans(x=numericDataScaled,centers=kOpt,iter.max=100)

classifOptKMeans <- kMeans_kOpt$cluster
classifOptKMeansClN <- as.factor(classifOptKMeans)
summary(classifOptKMeansClN)

som_gridOpt <- somgrid(xdim = 1, ydim=2, topo="rectangular")
som_modelOpt <- som(numericDataScaled,grid=som_gridOpt,rlen=1000,al-
pha=c(0.05,0.01), keep.data = TRUE)

classifOptSom <- som_modelOpt$unit.classif
classifOptSomClN <- as.factor(classifOptSom)
summary(classifOptSomClN)

BIC_modelOpt <- mclustBIC(numericDataScaled, G = c(2), prior = priorControl())
model_gmmOpt <-Mclust(numericDataScaled, x = BIC_modelOpt)
classifOptGmm <- model_gmmOpt$classification

classifOptGmmClN <- as.factor(classifOptGmm)
summary(classifOptGmmClN)

kmeansModel <- flexclust::kcca(numericDataScaled,k=2)
varImpKMeans <- FeatureImpCluster(kmeansModel,as.data.table(numericDataScaled))

m<-map.build(as.data.frame(numericDataScaled),labels=NULL,alpha=0.3,train=1000)
varImpSOM <- map.significance(m)

numericDataScaledDf <- as.data.frame(numericDataScaled)
res.hcpc <-HCPC(numericDataScaledDf, method = "ward", order=FALSE, consol =
```

```
FALSE, nb.clust = 2, metric = "euclidean")
table(res.hcpc$data.clust$clust)
varImpHAC <- res.hcpc$desc.var

varImpGMM <- clustvarsel(numericDataScaled,G=c(2),search="greedy",direction="b")


d = dist(numericDataScaled)
clustStatKMeansSOM <- cluster.stats(d,as.vector(classifOptKMeans),as.vector(clas-
sifOptSom))
clustStatKMeansSOM$vi

clustStatKMeansHAC <- cluster.stats(d, as.vector(classifOptKMeans),as.vector(clas-
sifOptHac))
clustStatKMeansHAC$vi

clustStatKMeansGMM <- cluster.stats(d, as.vector(classifOptKMeans),as.vector(clas-
sifOptGmm))
clustStatKMeansGMM$vi

clustStatSOMHAC <- cluster.stats(d,as.vector(classifOptSom),as.vector(classifOptHac))
clustStatSOMHAC$vi

clustStatSOMGMM <- cluster.stats(d,as.vector(classifOptSom),as.vector(clas-
sifOptGmm))
clustStatSOMGMM$vi

clustStatHACGMM <- cluster.stats(d,as.vector(classifOptHac),as.vector(classifOptGmm))
clustStatHACGMM$vi

vecMVIKMeans <- c(clustStatKMeansSOM$vi,clustStatKMeansHAC$vi,clustStat-
KMeansGMM$vi)
vecMVISOM <- c(clustStatKMeansSOM$vi,clustStatSOMHAC$vi,clustStat-
SOMGMM$vi)
vecMVIHAC <- c(clustStatKMeansHAC$vi, clustStatSOMHAC$vi, clustStatHAC-
GMM$vi)
vecMVIGMM <- c(clustStatKMeansGMM$vi,clustStatSOMGMM$vi,clustStatHAC-
GMM$vi)

minVI <- round(min(c(vecMVIKMeans,vecMVISOM,vecMVIHAC,vecMVIGMM)),digits
= 4)
maxVI <- round(max(c(vecMVIKMeans,vecMVISOM,vecMVIHAC,vecMVIGMM)),digits
= 4)

numMod <- 4

par(oma = c(1,5,1,2))
par(mar = c(1,1,3,1))

namesModelsKMeans <- c("SOM","HAC","GMM")

if(round(max(vecMVIKMeans), digits = 4)==maxVI){
  xtick <- round(vecMVIKMeans,digits = 4)
}else{
```

```
  xtick <- c(round(vecMVIKMeans,digits = 4),maxVI)
}
if(round(max(vecMVIKMeans), digits = 4)==maxVI){
  tmpDfKMeansMeila <- data.frame(cbind(namesModelsKMeans,vecMVIKMeans))
  tmpDfKMeansMeila <- tmpDfKMeansMeila[order(tmpDfKMeans-
Meila$vecMVIKMeans),]
  xtick <- round(as.numeric(as.vector(tmpDfKMeansMeila$vecMVIKMeans)),digits=4)
}else{
  tmpDfKMeansMeila <- data.frame(cbind(namesModelsKMeans,vecMVIKMeans))
  tmpDfKMeansMeila <- tmpDfKMeansMeila[order(tmpDfKMeans-
Meila$vecMVIKMeans),]
  xtick <- c(round(as.numeric(as.vector(tmpDfKMeansMeila$vecMVIKMeans)),dig-
its=4),maxVI)
}

par(cex.main = 1.4, cex.lab = 1.4)
barplot(round(as.numeric(as.vector(tmpDfKMeansMeila$vecMVIKMeans)), digits=4),
        horiz = T,
        names.arg = tmpDfKMeansMeila$namesModelsKMeans,
        las = 1,
        col = c("grey60","grey60","grey60"),
        border = NA,
        main = "Meila's Variation of Information\nK-Means Clustering vs others",
        xaxt="n",
        xlim = c(minVI,maxVI),
        cex.names = 1.4
)
axis(side=1, at=xtick, labels = xtick)

par(oma = c(1,5,1,2))
par(mar = c(1,1,3,1))

namesModelsSOM <- c("K-Means","HAC","GMM")

if(round(max(vecMVISOM), digits = 4)==maxVI){
  tmpDfSOMMeila <- data.frame(cbind(namesModelsSOM,vecMVISOM))
  tmpDfSOMMeila <- tmpDfSOMMeila[order(tmpDfSOMMeila$vecMVISOM),]
  xtick <- round(as.numeric(as.vector(tmpDfSOMMeila$vecMVISOM)),digits=4)
}else{
  tmpDfSOMMeila <- data.frame(cbind(namesModelsSOM,vecMVISOM))
  tmpDfSOMMeila <- tmpDfSOMMeila[order(tmpDfSOMMeila$vecMVISOM),]
  xtick <- c(round(as.numeric(as.vector(tmpDfSOMMeila$vecMVISOM)),dig-
its=4),maxVI)
}

par(cex.main = 1.4, cex.lab = 1.4)
barplot(round(as.numeric(as.vector(tmpDfSOMMeila$vecMVISOM)),digits=4),
        horiz = T,
        names.arg = tmpDfSOMMeila$namesModelsSOM,
        las = 1,
        col = c("grey60","grey60","grey60"),
        border = NA,
        main = "Meila's Variation of Information\nSOM Clustering vs others",
        xaxt="n",
```

```
            xlim = c(minVI,maxVI),
            cex.names = 1.4
)
axis(side=1, at=xtick, labels = xtick)

par(oma = c(1,5,1,2))
par(mar = c(1,1,3,1))

namesModelsHAC <- c("K-Means","SOM","GMM")

if(round(max(vecMVIHAC), digits = 4)==maxVI){
   tmpDfHACMeila <- data.frame(cbind(namesModelsHAC,vecMVIHAC))
   tmpDfHACMeila <- tmpDfHACMeila[order(tmpDfHACMeila$vecMVIHAC),]
   xtick <- round(as.numeric(as.vector(tmpDfHACMeila$vecMVIHAC)),digits=4)
}else{
   tmpDfHACMeila <- data.frame(cbind(namesModelsHAC,vecMVIHAC))
   tmpDfHACMeila <- tmpDfHACMeila[order(tmpDfHACMeila$vecMVIHAC),]
   xtick <- c(round(as.numeric(as.vector(tmpDfHACMeila$vecMVIHAC)),dig-
its=4),maxVI)
}

par(cex.main = 1.4, cex.lab = 1.4)
barplot(round(as.numeric(as.vector(tmpDfHACMeila$vecMVIHAC)),digits=4),
            horiz = T,
            names.arg = tmpDfHACMeila$namesModelsHAC,
            las = 1,
            col = c("grey60","grey60","grey60"),
            border = NA,
            main = "Meila's Variation of Information\nHAC Clustering vs others",
            xaxt="n",
            xlim = c(minVI,maxVI),
            cex.names = 1.4
)
axis(side=1, at=xtick, labels = xtick)

par(oma = c(1,5,1,2))
par(mar = c(1,1,3,1))

namesModelsGMM <- c("K-Means","SOM","HAC")

if(round(max(vecMVIGMM), digits = 4)==maxVI){
   tmpDfGMMMeila <- data.frame(cbind(namesModelsGMM,vecMVIGMM))
   tmpDfGMMMeila <- tmpDfGMMMeila[order(tmpDfGMMMeila$vecMVIGMM),]
   xtick <- round(as.numeric(as.vector(tmpDfGMMMeila$vecMVIGMM)),digits=4)
}else{
   tmpDfGMMMeila <- data.frame(cbind(namesModelsGMM,vecMVIGMM))
   tmpDfGMMMeila <- tmpDfGMMMeila[order(tmpDfGMMMeila$vecMVIGMM),]
   xtick <- c(round(as.numeric(as.vector(tmpDfGMMMeila$vecMVIGMM)),dig-
its=4),maxVI)
}
par(cex.main = 1.4, cex.lab = 1.4)
barplot(round(as.numeric(as.vector(tmpDfGMMMeila$vecMVIGMM)),digits=4),
            horiz = T,
            names.arg = tmpDfGMMMeila$namesModelsGMM,
```

```
        las = 1,
        col = c("grey60","grey60","grey60"),
        border = NA,
        main = "Meila's Variation of Information\nGMM Clustering vs others",
        xaxt="n",
        xlim = c(minVI,maxVI),
        cex.names = 1.4
)
axis(side=1, at=xtick, labels = xtick)
```
